# PAK4 and NAMPT as Novel Therapeutic Targets in Diffuse Large B-Cell Lymphoma, Follicular Lymphoma, and Mantle Cell Lymphoma

**DOI:** 10.3390/cancers14010160

**Published:** 2021-12-29

**Authors:** Husain Yar Khan, Md. Hafiz Uddin, Suresh Kumar Balasubramanian, Noor Sulaiman, Marium Iqbal, Mahmoud Chaker, Amro Aboukameel, Yiwei Li, William Senapedis, Erkan Baloglu, Ramzi M. Mohammad, Jeffrey Zonder, Asfar S. Azmi

**Affiliations:** 1 Departments of Oncology, Karmanos Cancer Institute, Wayne State University School of Medicine, Detroit, MI 48201, USA; khanh@karmanos.org (H.Y.K.); uddinh@wayne.edu (M.H.U.); balasubs@karmanos.org (S.K.B.); gk0888@wayne.edu (N.S.); marium.m.iqbal@uth.tmc.edu (M.I.); mchaker@dmc.org (M.C.); kameelo@karmanos.org (A.A.); liyi@karmanos.org (Y.L.); mohammad@karmanos.org (R.M.M.); zonderj@karmanos.org (J.Z.); 2 Karyopharm Therapeutics Inc., Newton, MA 02459, USA; wsenapedis@karyopharm.com; 3 Restorbio Inc., Boston, MA 02116, USA; ebaloglu@restorbio.com

**Keywords:** non-Hodgkin’s lymphoma, mantle cell lymphoma, p21 activated kinases, PAK4, NAMPT, NAD, KPT-9274

## Abstract

**Simple Summary:**

Non-Hodgkin’s lymphomas (NHL) are cancers of the white blood cells. While some NHL subtypes, such as Diffuse large B-cell lymphoma (DLBCL) and mantle cell lymphoma (MCL), grow and spread aggressively, others, like follicular lymphoma (FL), are indolent in nature. Irrespective of how fast they grow, all NHL subtypes can spread to other organs in the body if not treated. In this study, we have demonstrated that the targeted inhibition of p21-activated kinase 4 (PAK4) and nicotinamide phosphoribosyl transferase (NAMPT) in different NHL subtypes by a novel, orally bioavailable, dual inhibitor KPT-9274 can lead to energy depletion, inhibition of cell proliferation, and ultimately apoptosis. KPT-9274 treatment shows potent anti-tumor effects in DLBCL and MCL subcutaneous xenograft models and enhances mice survival in a systemic FL model. Therefore, this study demonstrates the potential of targeting PAK4 and NAMPT by a small molecule inhibitor KPT-9274 for NHL therapy.

**Abstract:**

Diffuse large B-cell lymphoma (DLBCL), grade 3b follicular lymphoma (FL), and mantle cell lymphoma (MCL) are aggressive non-Hodgkin’s lymphomas (NHL). Cure rates are suboptimal and novel treatment strategies are needed to improve outcomes. Here, we show that p21-activated kinase 4 (PAK4) and nicotinamide phosphoribosyl transferase (NAMPT) is critical for lymphoma subsistence. Dual targeting of PAK4-NAMPT by the Phase I small molecule KPT-9274 suppressed cell proliferation in DLBCL, FL, and MCL. Growth inhibition was concurrent with apoptosis induction alongside activation of pro-apoptotic proteins and reduced pro-survival markers. We observed NAD suppression, ATP reduction, and consequent cellular metabolic collapse in lymphoma cells due to KPT-9274 treatment. KPT-9274 in combination with standard-of-care chemotherapeutics led to superior inhibition of cell proliferation. In vivo, KPT-9274 could markedly suppress the growth of WSU-DLCL2 (DLBCL), Z-138, and JeKo-1 (MCL) sub-cutaneous xenografts, and a remarkable increase in host life span was shown, with a 50% cure of a systemic WSU-FSCCL (FL) model. Residual tumor analysis confirmed a reduction in total and phosphorylated PAK4 and activation of the pro-apoptotic cascade. This study, using various preclinical experimental models, demonstrates the therapeutic potential of targeting PAK4-NAMPT in DLBCL, FL, and MCL. The orally bioavailable, safe, and efficacious PAK4-NAMPT dual inhibitor KPT-9274 warrants further clinical investigation.

## 1. Introduction

Non-Hodgkin’s lymphoma (NHL) is a heterogeneous group of neoplastic proliferations of B-cells with distinct clinicopathologic, immunotypic, and genetic features. Apart from Burkitt’s lymphoma, other aggressive B-cell NHLs include diffuse large B-cell lymphoma (DLBCL), grade-3b follicular lymphoma (FL), and mantle cell lymphoma (MCL). Chemo-immunotherapy is the standard of care for all these aggressive lymphomas in the first-line setting. Although the 5-year survival rate for DLBCL is approximately 60%, up to 50% do relapse eventually or become refractory to frontline treatment [1]. The less common variant of diffuse FL has a five-year survival rate of around 50% [2]. MCL are generally more aggressive, and most studies, even with intensive treatment regimens, failed to demonstrate a plateau in the survival curve on long-term follow-up [3]. Despite rationally applied molecularly driven combined chemo-immunotherapeutic approaches, prognosis remains dismal, especially for the relapsed/refractory (R/R) aggressive NHLs calling for novel treatment strategies. 

Deregulated cellular energetics, mainly due to the high metabolic demand of rapidly proliferating cancer cells, is often observed in solid tumors. However, the analysis of such altered energetics in hematological malignancies is lacking. Since the cancer cells rely on nicotinamide adenine dinucleotide (NAD)-dependent enzymes for their increased metabolic demand, the NAD turnover rate is higher than in normal cells. The chief sources for NAD production are nicotinic acid, nicotinamide, and tryptophan [4]. Three pathways can regenerate NAD: (a) catabolism of tryptophan, a multi-step process occurring predominantly in the liver [5]; (b) NAM salvage via the NAMPT pathway [6]; or (c) salvage of exogenous niacin (vitamin B3, nicotinic acid, or NA) through the NAPRT1 pathway [7,8]. NAD^+^ supply mainly relies on the recycling pathway in physiological states [9]. Its salvage and recycling synthesis are regulated by nicotinamide phosphoribosyl transferase (NAMPT) in a rate-limiting step [10]. It is not surprising to note that several tumor cells have a greater expression of NAMPT [11]. In addition, ~20–25% of all cancer types are NAPRT1 deficient (mainly through hypermethylation of its promoter). Various NAMPT inhibitors, such as GMX1778 and APO866 (FK866), have been developed [12] and found to be efficacious in several tumors in vitro and in animal systems [13,14,15,16]. 

The mitogen-activated kinase (MAPK) pathways are almost universally deregulated in cancer. Increased levels of activated p38 MAPK (phospho-p38 MAPK) have been correlated with follicular lymphoma and other malignancies [17,18,19,20]. The p21-activated kinases (PAK) are a part of the serine/threonine kinases family of STE20 [21]. PAK4 promotes cell proliferation via the c-Src/EGFR/cyclin D1 pathway and cell survival through the TNF-α receptor complex [22]. Studies show PAK4 overexpression in tumor cell lines of the ovary, pancreas, colon, prostate, lung, and leukemia compared to their respective normal cell lines [23]. More importantly, NHL cell lines demonstrated increased expression of PAK4 mRNA compared to normal peripheral lymphocytes [24]. 

Given the role of NAMPT and PAK4 in lymphoma biology, dual inhibition of NAMPT-PAK4 appears to be a promising therapeutic strategy. KPT-9274 is a first-in-class orally bioavailable dual inhibitor of NAMPT and PAK4. It has shown activity against a range of solid and hematological malignancies by reducing growth via modulating PAK4, Wnt/β-catenin signaling, and inhibiting NAD synthesis [25]. In this report, we demonstrate the anti-proliferative activity of KPT-9274 and analogs in NHL models in vitro and in vivo. These results strengthen our phase I study design to evaluate safety, tolerability, and anti-tumor activity in patients with advanced solid malignancies or NHL treated with KPT-9274 ± niacin (NCT02702492). 

## 2. Materials and Methods

### 2.1. Cell Lines, Reagents, and Culture Conditions

WSU-FSCCL, representing follicular small cleaved cell lymphoma, generated from a leukemic variant of transformed follicular lymphoma with rearranged bcl2 and c-Myc and WSU-DLCL2 representing diffuse large B-cell lymphoma, have been developed by our laboratory at Wayne State University [26]. All other cells lines were purchased from ATCC. WSU-DLCL2 and WSU-FSCCL cells were authenticated using Short Tandem Repeat (STR) profiling at the Wayne State University, Karmanos Cancer Institute, Biobanking Core. Both cell lines were maintained in RPMI 1640 medium supplemented with 10% fetal bovine serum (Hyclone Laboratories, Logan, UT, USA) and 1% penicillin–streptomycin (Invitrogen, Carlsbad, CA, USA), at 37 °C in a humidified incubator with 5% CO_2_. Primary antibodies for Bcl-2, PARP, cleaved PARP, cleaved Caspase 3, CDK4, and β-actin, were purchased from Cell Signaling (Danvers, MA, USA). Antibody for CDK2 and all secondary antibodies were obtained from Sigma (St. Louis, MO, USA). KPT-8752 and KPT-9274 were prepared utilizing the synthetic scheme and experimental procedures described in WO2015003166 (US 2016/0368904 US Patent: US20160368904A1) [27]. PAK4 specific inhibitor PF-3578309 [28] and NAMPT specific inhibitor FK-866 [29] were purchased from SelleckChem (Houston, TX, USA). All inhibitors were dissolved in dimethyl sulfoxide (DMSO). The drug control for inhibitor experiments was cell culture media containing 0.1% DMSO. 

### 2.2. Immunohistochemistry of Tissue Microarray

IHC automated quantification using Visiopharm was performed by US Biomax Inc. (Derwood, MD, USA) on diffuse large B-cell lymphoma tissue array and normal lymph node tissue. Anti-Pak4 antibody was obtained from Abcam (Waltham, MA, USA) and used at a dilution of 1:800 for staining the tissue microarray. The staining score for IHC was calculated by a semi-quantitative assessment of both the staining intensity (graded as: –, negative; +, weak; ++, moderate; or +++, strong) and the percentage of positive cells over the total cells. The calculation formula was: Staining score = (% stained cells at 1+) × 1 + (% stained cells at 2+) × 2 + (% stained cells at 3+) × 3.

### 2.3. Cell Viability Determined by Trypan Blue Assay

Cells were seeded at a density of 2 × 10^5^ viable cells/mL in 24-well plates (Costar, Cambridge, MA, USA). KPT-9274 was added at increasing concentrations (0–500 nM), diluted from a 10 μM stock. After 72 h of treatment, cell viability was determined by a Trypan Blue dye exclusion test (Trypan blue (0.4%), Sigma-Aldrich, St. Louis, MO, USA). For combination studies, WSU-DLCL2 and WSU-FSCCL cells were exposed to KPT-9274 (75 nM for WSU-DLCL2 and 10 nM for WSU-FSCCL) in the absence or presence of cyclophosphamide, oncovin, or adriamycin (IC_25_ or IC_50_) for 72 h followed by Trypan blue viability analysis. The results were plotted as means ± SEM of three separate experiments using three determinations per experiment for each experimental condition.

### 2.4. Quantification of Apoptosis by 7-Amino-Actinomycin D (7-AAD)

Viable, non-viable, and apoptotic cells were detected using 7-AAD assay (Calbiochem-Novabiochem, La Jolla, CA, USA) in flow cytometric analysis. Briefly, NHL cells (WSU-DLCL2 and WSU-FSCCL) were seeded in 6-well culture plates at a density of 2 × 10^5^ viable cells/mL and treated with KPT-9274 at or above IC_50_. Postive controls PF-3758309 (a PAK4 specific inhibitor), or FK866 (a NAMPT inhibitor) were also used to treat the cells. Seventy-two hours later, cells were collected, counted, washed with PBS, and stained with 7-AAD at the manufacturer’s suggested dilution. Cells were then analyzed on a FACScan (Becton Dickinson, Mountain View, CA, USA). Data on 20,000 cells were acquired and processed using Lysys II software (Becton Dickinson). Scattergrams were generated by combining forward light scatter with 7-amino-actinomycin D fluorescence.

### 2.5. ATP and NAD Analyses

Total ATP and NAD levels were evaluated using luminescent assays (Promega, Madison, WI, USA) according to the manufacturer’s protocol. In brief, WSU-DLCL2 and WSU-FSCCL cells were grown in opaque walled 96-well plates and exposed to increasing KPT-9274 concentrations for indicated periods. ATP levels were determined by adding CellTiter-Glo Luminescent cell viability reagent (catalog number G7571) to each well and incubated for 30 min. NAD/NADH levels were obtained by adding NAD/NADH-Glo reagent (catalog number G9072) and incubated for 30 min. Luminescent signal was then detected using SpectraMax i3x and SoftmaxPro software. 

### 2.6. Western Blotting

WSU-DLCL2 and WSU-FSCCL cells (1 × 10^6^) were grown in T75 flasks and exposed to indicated concentrations of KPT-9274 for 72 h, followed by extraction of total protein for western blot analysis using previously described methods [30]. 

### 2.7. Development of Subcutaneous DLBCL Xenograft Model

Using established WSU-DLCL2 tumor fragments (each at about 50–60 mg), 12 female ICR SCID mice (Taconic laboratories) were unilaterally and subcutaneously transplanted with the fragments. When confirmed tumors became palpable (about one week later), the animals were randomly assigned to two different cohorts (Untreated and KPT-9274 treated). The maximum tolerated dose (MTD) of KPT-9274 in ICR mice was previously determined to be above 200 mg/kg. Mice were gavaged orally (twice daily for five days with two days off for 3–4 weeks) with 150 mg/kg KT-9274. Both groups were followed for measurement of subcutaneous tumors, changes in body weight, and other potential side effects of the drug. Tumors were measured by a caliper at least twice weekly. Tumor volume (mm^3^) was calculated using the standard formula: (*A* × *B*^2^)/2, where *A* and *B* are the tumor length and width (in mm). To avoid discomfort, and in keeping with our IACUC guidelines, animals were euthanized when their total tumor burden reached 5–10% of their body weight, about 2000 mg. Random tumor collection occurred at the time of euthanasia. All studies involving mice were performed under the Animal Investigation Committee-approved protocol (# 15-11-023).

### 2.8. Isolation of Tumor Tissue Proteins and Western Blot Analysis

The expression of p-PAK4 and Caspase 9 was analyzed from collected WSU-DLCL2 tumors using western blot. Once collected, tumors were snap-frozen and later homogenized using a tissue lyser (QIAGEN) in T-PER buffer (PIERCE) containing protease and phosphatase inhibitors. Insoluble debris was removed by centrifugation at 20,000 rpm for 10 min. Protein concentration was determined using the Pierce BCA protein assay kit, and equal amounts of protein were prepared in SDS sample buffer. The samples were boiled for 10 min and subjected to SDS-PAGE. The proteins were then transferred to a nitrocellulose membrane using the iBlot system. The membrane was then blocked for 30 min using Odyssey blocking buffer. The blots were then probed overnight for the protein of interest and washed three times with PBS-T for 15 min. Secondary antibodies were added for 30 min, and three additional washes were performed. The blots were then visualized using the Odyssey imaging system.

### 2.9. RNA Isolation and mRNA Real-Time RT-qPCR

Total RNAs from mouse tumors were extracted and purified using the RNeasy Mini Kit and RNase-free DNase Set (QIAGEN, Valencia, CA, USA) following the protocol provided by the manufacturer. The expression levels of Pak4, Bcl-2, β-catenin, and Caspase 9 in the mouse tumor tissues were analyzed by real-time RT–qPCR using a High Capacity cDNA Reverse Transcription Kit and SYBR Green Master Mixture from Applied Biosystems (Waltham, MA, USA). The sequences of primers used are listed in Table 1. The qPCR was initiated by 10 min at 95 °C before 40 thermal cycles, each of 15 s at 95 °C and 1 min at 60 °C in a StepOnePlus real-time PCR system (Applied Biosystems). Data were analyzed according to the comparative Ct method and were normalized by actin and 18S rRNA expression in each sample.

### 2.10. Development of Systemic FL Xenograft Model

All animal studies were conducted under WSU IACUC approved protocol (# 15-11-023), and animal care was in accordance with the institute’s guidelines. Using our established and authenticated WSU-FSCCL cell line, 1 × 10^7^ cells were injected intravenously through the tail veins of 11 female ICR mice. One-week post-inoculation, mice were randomly separated into two groups: untreated and KPT-9274-treated. The dose and schedule mentioned above were used for this experiment. Seventy-two hours prior to and three weeks after the last KPT-9274 treatment, blood was drawn from the tail vein of random mice using a 26 G needle and blood smears were prepared on micro slides. Once dried, the slides were stained with Tetrachrome stain (Sigma-Aldrich, St. Louis, MO, USA), and pictures were taken. Mice were followed for 150 days post-inoculation. At this time point, all remaining live mice without any signs of morbidity were considered cured. Another experiment was conducted using the same model and methodology with 10 mice to test KPT-8752. KPT-8752 was used at 60 mg/kg orally twice daily for five days with two days off for 2–3 weeks.

### 2.11. Development of Subcutaneous MCL Xenograft Models

Z-138 (ATCC # CRL-3001) mantle cell lymphoma cells were grown in IMEM medium supplemented with 10% Horse Serum, 1% penicillin and streptomycin, and 2mM L-Glutamine. Thirty-two nu/nu mice were inoculated subcutaneously in the left flank with 1 × 10^7^ Z-138 cells. Mice were allocated to four groups of eight mice each. Mice were treated with either placebo, niacin (30 mg/kg PO BID), KPT-9274 (200 mg/kg PO BID), or a combination of KPT-9274 and niacin. 

JEKO-1 (ATCC # CRL-3006) mantle cell lymphoma cells were grown in IMEM medium supplemented with 10% fetal bovine serum, 1% penicillin and streptomycin, and 2mM L-Glutamine. Thirty-two NOD-SCID mice were inoculated subcutaneously in the left flank with 1 × 10^7^ JEKO-1 cells. Mice were allocated into four groups of eight mice each. Mice were treated with placebo, KPT-9274 (200 mg/kg PO QoDX3), niacin (100 mg/kg PO QoDX3), or a combination of KPT-9274 with niacin. 

Tumors were measured once every two days with micro-calipers, and tumor volume was calculated as (length × width × width)/2.

### 2.12. Residual Tumor Marker Analysis

The expression of different PAK4 related markers was detected in histological sections of Z-138 tumor xenografts. Sections were cut at 4 µm, standard hematoxylin (Richard-Allan) and eosin (Richard-Allan) staining were performed. IHC assays were performed on a Biogenex i6000 automated stainer using a Cell Marque Hi-Def Polymer Detection Kit. The sections were incubated with anti-PAK4, GEFH-1, vinculin, paxillin, cyclin D1, and Ki67 overnight at room temperature in a humidified atmosphere followed by a 30-min incubation with a secondary antibody. ApopTag kit (EMD Millipore) was used to mark apoptotic cells as per the manufacturer’s instructions. 

### 2.13. Statistical Analysis

Wherever appropriate, the data were subjected to a Student’s *t*-test using GraphPad Prism software (La Jolla, CA, USA). *p* < 0.05 was considered statistically significant. Statistical differences between animal treatment groups were determined using Mann–Whitney Rank Sum or ANOVA tests with a critical value of 0.05.

## 3. Results

### 3.1. PAK4-NAMPT Is Essential for Lymphoma Cell Survival

**Figure 1:**
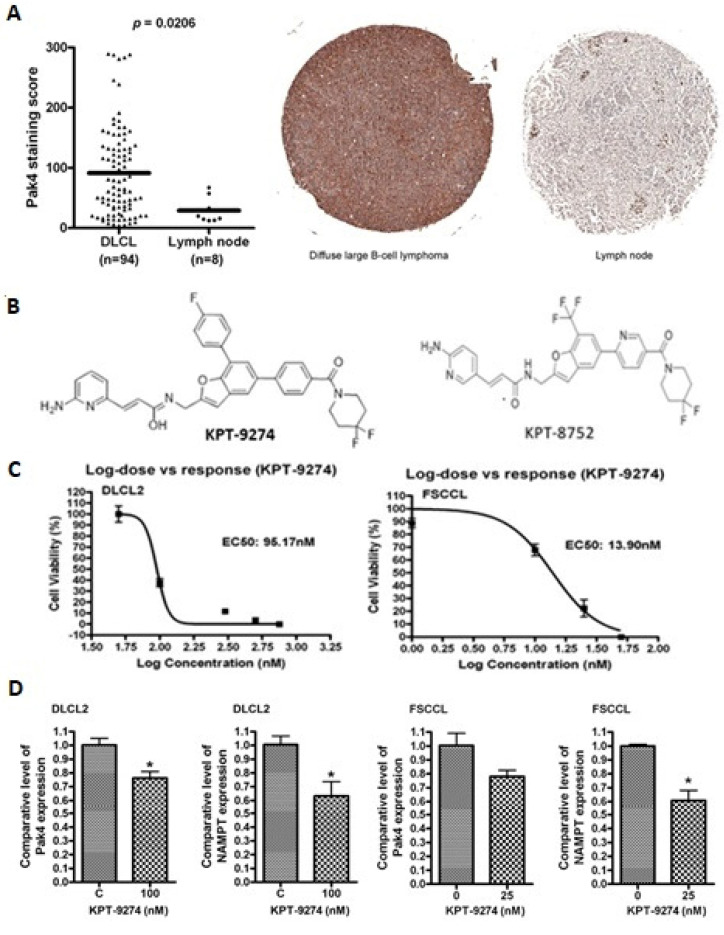
PAK4-NAMPT is critical for WSU-DLBCL and WSU-FSCCL cell sustenance. (**A**) IHC of tissue microarray of NHL biopsies (*n* = 94) compared to normal lymph node biopsies (*n* = 8), showing enhancement in PAK4 expression. (**B**) Structures of KPT-9274 and KPT-8752. (**C**) To assess the effect of PAK4 inhibition on NHL, WSU-DLCL2 and WSU-FSCCL cells were seeded in duplicates in 24-well plates and exposed to different concentrations of KPT-9274 for 72 h. At the end of the treatment period, cells were counted using Trypan Blue to determine cell viability. Data is representative of three independent experiments. (**D**) WSU-DLCL2 and WSU-FSCCL cells were grown in 6-well plates and exposed to indicated concentrations of KPT-9274 for 24 h. At the end of the treatment period, RNA was isolated, and qRT–PCR was performed as described in the Methods section. Graphs are representative of two independent experiments. * *p* < 0.05.

To evaluate the expression of PAK4 in lymphomas, IHC on tissue microarrays was performed. As seen in Figure 1A, PAK4 was highly expressed in NHL (Diffuse large B-cell lymphoma) primary tumor tissue (*n* = 94) vs. normal lymph nodes (*n* = 8). Table S1 gives the pathology report for these tissue microarrays. In addition, we performed PAK4 IHC staining across different B-cell lymphoma specimens (3 DLBCL (2 ABC and 1 GC), 1 MCL (high grade) and 3 low-grade NHLs) with an established pathology report from our institutional biorepository (Karmanos Cancer Institute Biospecimen Collection Protocol # 2015-030). There was a strong correlation noted between the proliferation index (Ki67) and the PAK4 staining across different high-grade lymphomas. Higher PAK4 staining scores were found to be characteristic for high grade non-Hodgkin’s lymphomas including a specimen of mantle cell lymphoma with a Ki67 of 80%. Contrastingly, PAK4 staining in a sample of small lymphocytic lymphoma and two low-grade NHLs had a lesser score (Figure S1).

Lymphoma cell exposure to a low nanomolar concentration of dual PAK4-NAMPT inhibitor KPT-9274 (structure shown in Figure 1B) led to inhibition of cell proliferation (Figure 1C). KPT-9274 inhibited growth with an EC_50_ of 95.17 nM in WSU-DLCL2 and 13.9 nM in WSU-FSCCL cells. To assess the effect of KPT-9274 on normal cells, peripheral blood mononuclear cells (PBMC) isolated from the blood of a healthy, non-smoking donor (the lead author) were treated with different concentrations of KPT-9274 (0–750 nM) for 72 h. KPT-9274 treatment does not result in any significant loss of cell viability at doses as high as 750 nM (Figure S2). The EC_50_ of KPT-9274 for PBMC is determined to be 1836 nM, which is much higher as compared to that for WSU-DLCL2 and WSU-FSCCL cells. KPT-9274 activity is not restricted to lymphoma models alone. Several studies published by our group and those of others have established the cancer cell selectivity of these agents in which high micromolar EC_50_s were observed in normal peripheral lymphocytes and normal human pancreatic ductal epithelial cells (>500 nM) [31,32,33]. Results from qRT–PCR showed that KPT-9274 treatment resulted in the reduction of PAK4 and NAMPT expression in WSU-DLCL2 and WSU-FSCCL cells (Figure 1D). These results strengthen our hypothesis that PAK4-NAMPT is critical for lymphoma cell survival, and their targeted inhibition could be an effective strategy against these challenging diseases. 

### 3.2. PAK4-NAMPT Inhibition Induces Apoptosis

**Figure 2:**
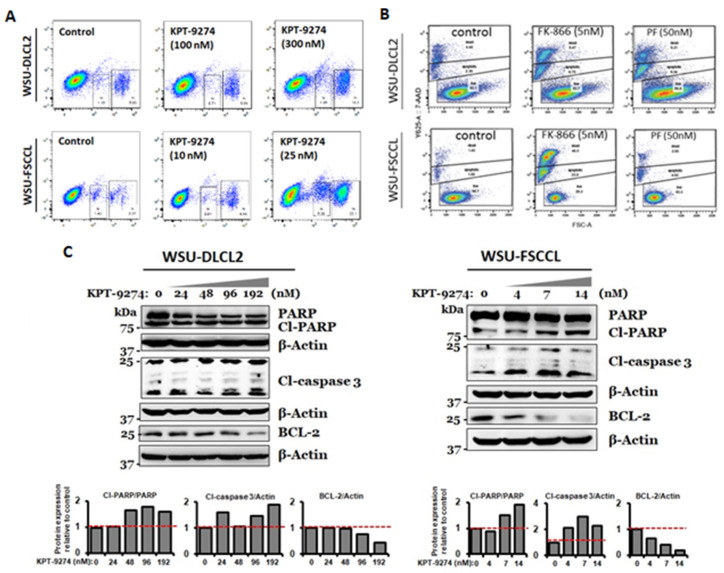
PAK4-NAMPT dual inhibition induces apoptosis in WSU-DLCL2 and WSU-FSCCL cellular models. NHL cells were grown in 24-well plates at a density of 200,000 cells per well and exposed to indicated doses of KPT-9274 (**A**) or +ve controls (**B**) for 72 h. At the end of the treatment period, cells were centrifuged, and the supernatant was discarded. Cells were suspended in 7-AAD solution followed by analysis for apoptosis. 7-AAD graphs are representative of two independent experiments. (**C**) WSU-DLCL2 and WSU-FSCCL cells were treated with indicated concentrations of KPT-9274 for 72 h. At the end of the treatment period, cells were centrifuged, and protein was isolated using standard procedures. Equal amounts of proteins were resolved on 10–12% polyacrylamide gels, followed by western blotting. The blots were probed for apoptosis pathway proteins. β-actin was used as a loading control. Change in protein expression was quantified by measuring relative band intensities using NIH ImageJ 1.5Oi software.

To verify whether cell proliferation inhibition consequently led to apoptosis, 7-AAD apoptosis assay was performed. Exposure to KPT-9274 at low nanomolar doses resulted in significant induction of apoptosis in two NHL cell lines (Figure 2A). The apoptosis induction was more significant in the WSU-FSCCL cell line compared to WSU-DLCL2. However, both these cell lines responded to the drug in the low nanomolar range. Similar results were obtained with commercially available PAK4 specific inhibitor PF-3578309 and NAMPT specific inhibitor FK-866 used as positive controls (Figure 2B). We next evaluated the change in expression of pro-survival and pro-apoptotic markers in WSU-DLCL2 and WSU-FSCCL cells upon KPT-9274 treatment. Exposure of the lymphoma cells to nanomolar doses of KPT-9274 led to a significant reduction in the expression of Bcl-2 (pro-survival marker) and enhanced expression of pro-apoptotic markers, such as cleaved-Caspase-3 and cleaved-PARP (Figure 2C and Figure S4). In addition, we also performed western blot analysis for cell cycle markers and observed a reduction in the expression of CDK4 and CDK2, indicating cell cycle arrest, as a result of treatment with increasing doses of KPT-9274 (Figure S3). These results further reinforce our hypothesis that targeted inhibition of PAK4-NAMPT could be a feasible therapeutic approach against lymphomas.

### 3.3. Impact of KPT-9274 on Cellular ATP and NAD Pool

**Figure 3:**
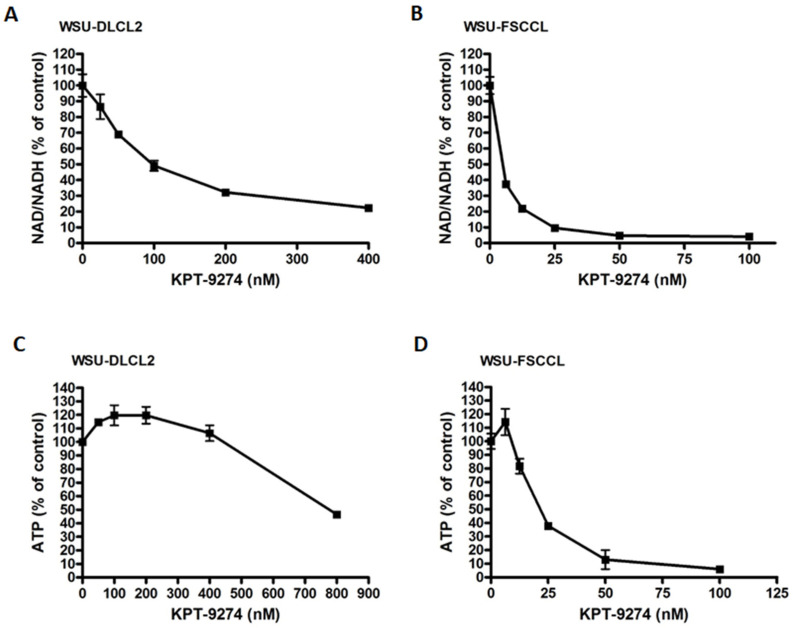
KPT-9274 causes cell metabolic collapse. (**A**,**B**) WSU-DLCL2 and WSU-FSCCL cells were seeded in opaque 96-well plates at a density of 20,000 cells per well and incubated overnight. The cells were then treated with KPT-9274 at different concentrations for 24 h. NAD/NADH assay was performed using a NAD/NADH-Glo assay kit (Promega) according to the manufacturer’s manual. The levels of NAD/NADH were quantified by a BioTek SynergyHT Plate Reader using a luminescence module. (**C**,**D**) WSU-DLCL2 and WSU-FSCCL cells were seeded in opaque 96-well plates at a density of 20,000 cells per well and incubated overnight. The cells were then treated with KPT-9274 at different concentrations for 48 h. An ATP assay was conducted using the CellTiter-Glo assay kit (Promega) according to the manufacturer’s manual. The level of ATP was quantified by a BioTek SynergyHT Plate Reader using a luminescence module. Graphs are representative of two independent experiments.

KPT-9274 is a dual inhibitor of PAK4 and NAMPT. Since NAMPT catalyzes the rate limiting step in the nicotinamide adenine dinucleotide (NAD) pathway, inhibiting the NAD pathway may lead to ATP depletion and cell death. In fact, NAMPT inhibition has been shown to suppress cellular NAD and ATP [34]. Therefore, we wanted to determine whether KPT-9274 can inhibit NAD production and ATP depletion in cancer cells. The NAD/NADH-Glo assay was performed to assess the effect of NAMPT inhibition on NAD status in WSU-DLCL2 and WSU-FSCCL cells. The effect of NAMPT inhibition on ATP was also evaluated by ATP CellTiter-Glo assay. NAD/NADH decreased in both cell lines with increasing KPT-9274 concentrations (Figure 3A,B). ATP levels also decreased significantly with escalating concentrations of KPT-9274 (Figure 3C,D).

### 3.4. KPT-9274 Chemotherapy Combination Studies

**Figure 4:**
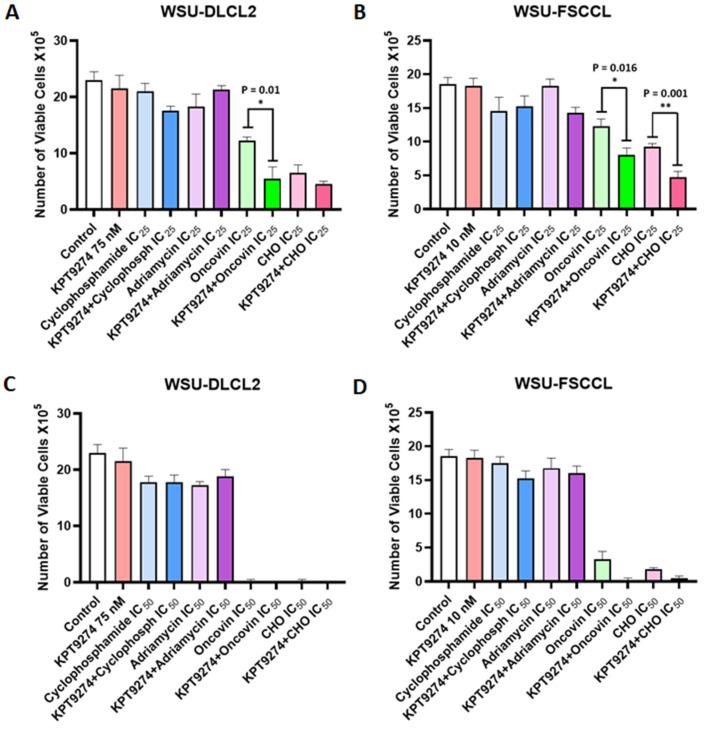
KPT-9274 chemotherapy combination. (**A**,**B**) WSU-DLCL2 and WSU-FSCCL cells were exposed to either vehicle (DMSO); 75 nM of KPT-9274 (for WSU-DLCL2) and 10 nM (for WSU-FSCCL); cyclophosphamide (IC_25_); Adriamycin (IC_25_); oncovin (IC_25_) alone or in combination for 72 h, followed by assessment of cell viability using Trypan Blue assay. (**C**,**D**) WSU-DLCL2 and WSU-FSCCL cells were exposed to either vehicle (DMSO); 75 nM of KPT-9274 (for WSU-DLCL2) and 10 nM (for WSU-FSCCL); cyclophosphamide (IC_25_); Adriamycin (IC_25_); oncovin (IC_25_) alone or in combination for 72 h followed by assessment of cell viability using Trypan Blue assay. Plots are representative of two independent experiments. * *p* < 0.05, ** *p* < 0.005.

We further evaluated whether KPT-9274 could enhance the activity of standard-of-care chemotherapeutics. For this, we selected CHO (Cyclophosphamide, Doxorubicin (Adriamycin), and Vincristine (Oncovin)), which is part of the chemotherapy backbone for NHL treatment (CHOP). Exposure to a low dose (IC_25_) of Oncovin or the triple combination (CHO) led to inhibition of proliferation of WSU-DLCL2 and WSU-FSCCL cell lines. However, when combined with KPT-9274, there was statistically significant and superior inhibition of NHL cell proliferation. The combination of KPT-9274 with single agent Oncovin was considerably effective. Furthermore, when used at IC_50_ concentrations, the chemotherapeutics drastically reduced the growth of the two cell lines (Figure 4C,D). These results establish KPT-9274 as a potential synergist to enhance the efficacy of standard-of-care chemotherapeutics in NHL.

### 3.5. Preclinical Anti-Tumor Efficacy Trial of KPT-9274 in Lymphoma Xenografts

**Figure 5:**
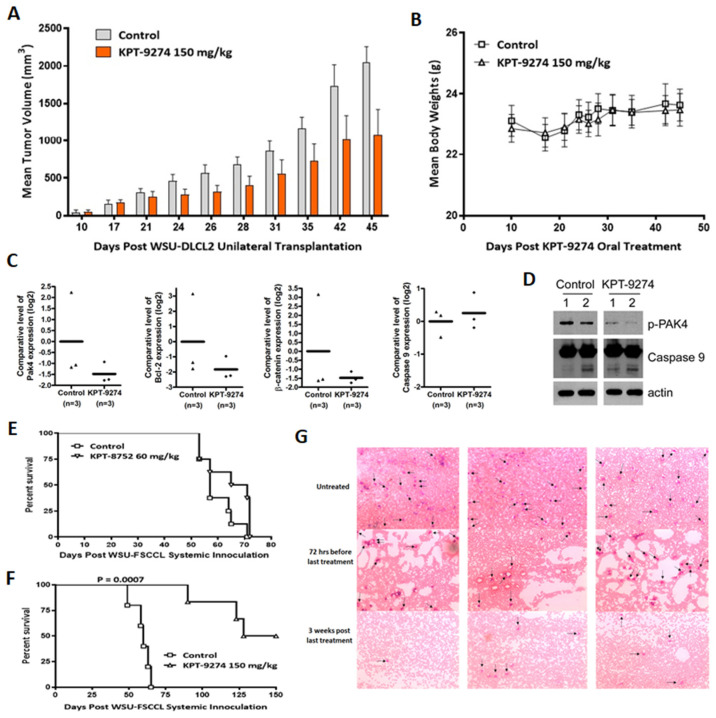
Anti-tumor efficacy of KPT-9274 in DLBCL and FL xenograft models. (**A**) WSU-DLCL2 fragments were engrafted in the flank of ICR SCID mice. On day 10 post unilateral transplantation, mice were administered KPT-9274 orally at a dose of 150 mg/kg daily for three weeks. Tumor volumes were recorded at indicated days using standard procedures. (**B**) Mice body weight was recorded every 3–5 days over the treatment period. Tumors were harvested at the end of the treatment and divided into two parts (one for RNA isolation and the second for protein isolation and western blotting). (**C**) qRT–PCR was performed on three control and three KPT-9274 treatment mice tumors using standard procedures. (**D**) Protein was isolated from two control and two KPT-9274 treated animals and resolved on 10% polyacrylamide gel electrophoresis, and western blotting was performed according to standard procedures. (**E**,**F**) WSU-FSCCL cells were injected systemically via the tail vein. After 7 days, mice were exposed to KPT-8752 or KPT-9274 at indicated doses. Mice were followed until death or 150 days post-inoculation. (**G**) Blood smear analysis of systemically xenografted mice treated with KPT-9274. Arrows point to the WSU-FSCCL cells among other blood cells. Images were captured at 100× magnification. Please add scale bar or magnification for Figure 5G.

In vivo studies were performed to assess the effect of PAK4-NAMPT inhibition on NHL tumor xenografts. KPT-9274 treatment of mice subcutaneously transplanted with WSU-DLCL2 fragments resulted in an approximately 50% reduction in tumor volumes (Figure 5A) without any significant change in body weight (Figure 5B). A paired one tailed *t*-test found the reduction in tumor volumes to be statistically significant (*p* = 0.0059). qRT–PCR on RNA from residual tumors (*n* = 3) showed significant inhibition of PAK4 alongside suppression of pro-survival protein Bcl-2 and the PAK4 target β-catenin. Furthermore, we also observed activation of caspase 9, which indicates a role for apoptosis induction in tumor reduction (Figure 5C). The qRT–PCR results were further corroborated with the western blotting data in which we observed a decrease in p-PAK4 alongside enhancement in cleaved caspase 9 (Figure 5D and Figure S4). 

In the WSU-FSCCL systemic xenograft model, animal survival inversely correlates with tumor activity. Treatment with KPT-8752, an analog of KPT-9274, resulted in a marginal to no improvement in survival compared to the control (Figure 5E). KPT-9274 treatment, however, improved animal survival drastically (>150 day increase in host life span compared to control and 3/6 cured mice). Enhanced survival in the KPT-9274 treated group was statistically significant (*p* = 0.0007) compared to untreated animals (Figure 5F). After day 150, the remaining mice were dissected to confirm cures. Grossly, there was no evidence of disseminated disease, especially to the brain (leptomeningeal infiltration), where historically, all WSU-FSCCL injected animals have succumbed. The effects of the KPT-9274 treatment were long-lasting, as evident by the prolonged mice survival. Blood smears show dramatically decreased WSU-FSCCL cells in the blood of KPT-9274 treated mice three weeks post last treatment, indicating the efficacy of the treatment (Figure 5G).

**Figure 6:**
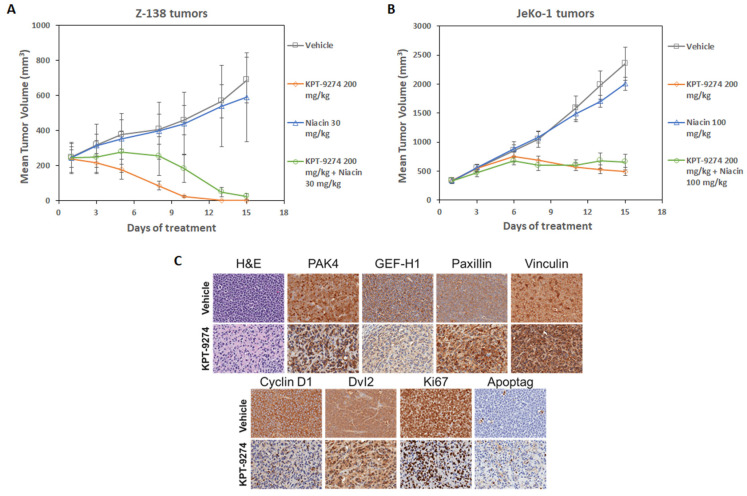
Anti-tumor effects of KPT-9274 in MCL xenograft model. (**A**,**B**) Treatment with KPT-9274 leads to tumor volume reduction in mice bearing Z-138 and JeKo-1 subcutaneous tumor xenografts. Co-dosing KPT-9274 with niacin at the indicated doses does not result in loss of efficacy in both the xenograft models. (**C**) Immunohistochemical analysis of Z-138 residual tumor tissues for H&E, PAK4, GEF-H1, paxillin, vinculin, cyclin D1, Dvl2, ki67, and apoptag. Images were captured at 200× magnification. Please add scale bar or magnification for Figure 6C.

The impact of niacin co-dosing with KPT-9274 was evaluated on tumor growth using the Z-138 MCL tumor xenograft model in nude mice. Tumor bearing mice were treated with vehicle, KPT-9274 given twice daily at 200 mg/kg, niacin at 30 mg/kg, or a combination of KPT-9274 and niacin. All groups that received KPT-9274 treatment showed dramatic reductions in tumor size, while the vehicle control and niacin groups showed rapid increases in tumor volume. Both KPT-9274 and combination treatment groups showed high levels of statistical significance in the reduction of tumor growth (*p* = 0.0002); however, there were no statistically significant differences between these two groups (Figure 6A). Similarly, in another MCL xenograft model, KPT-9274 treatment resulted in statistically significant reduction in JeKo-1 tumors (*p* = 0.0059). Co-dosing the tumor bearing mice with niacin, did not rescue the tumor growth suppression induced by KPT-9274 treatment (Figure 6B). No animal deaths were seen in this study. We also performed a detailed immunohistochemical analysis of the KPT-9274 treated Z-138 residual tumors. Our results (Figure 6C) show that KPT-9274 treatment caused a dramatic reduction in PAK4, GEF-H1, paxillin, and vinculin. Reduced expression of cyclin D1, Dvl2, and Ki67 was also observed. Simultaneously, we also observed enhancement in apoptosis in the treated tumors using Apoptag staining. These results strongly demonstrate the anti-tumor potential of KPT-9274 in lymphoma models and indicate their utility in these threatening diseases.

## 4. Discussion

Aggressive variants of NHLs can be inherently resistant to conventional frontline therapies and, when relapsed, have poor outcomes. In this paper, we demonstrate that PAK4-NAMPT dual inhibition results in the suppression of cell growth and tumor growth reductions in DLBCL, FL, and MCL models. Our studies bring forward two novel therapeutic targets in these difficult-to-treat lymphoid diseases. 

NAD is an essential metabolite for cell survival. There are three pathways for NAD production, namely, the NAMPT-dependent and the NAPRT1-dependent salvage pathway, which require nicotinamide (NAM) and niacin, respectively, and de novo synthesis from tryptophan. Cancer cells rely more upon NAMPT for NAD production due to the ready pool of NAM. Hence, low niacin levels, lack of de novo synthesis genes, and selective silencing of NAPRT1 in cancer cells inhibiting the NAMPT pathway create the necessary vulnerability to inhibit cancer cell growth. In contrast, niacin supplementation can rescue normal cells from the toxic effects of NAMPT inhibition [35]. As expected, the NAD/NADH ratio decreased in all cell lines when treated with KPT-9274, ultimately leading to a decreased ATP level. We have shown KPT-9274 to be an effective treatment of NHL, as it reduced cell viability, induced apoptosis, and decreased the production of NAD in the cancer cells. These results were directly proportional to the drug dosage. 

PAK4 is overexpressed in many solid and liquid malignancies, and its amplification indicates aggressiveness and poor prognosis [36]. PAK4 has several functions at the cellular level, which are associated with oncogenesis. The chromosomal region coding for PAK4, 19q13.2, is amplified in many aggressive cancers and therefore overexpressed in cancer cell lines, while it is undetectable in normal cells [37]. In cancerous cells, PAK is activated with mutated Rac [38]. The major functions of PAK include cell proliferation stimulation, cell motility, and survival. Once deregulated, as in tumorigenesis, PAK signaling induces cell transformation, angiogenesis, epithelial to mesenchymal transformation, increased proliferation, and dysregulation of the actin cytoskeleton, resulting in cell motility and invasion [39,40]. 

PAK4 inhibition by RNAi or small molecule drugs has been shown to dramatically decrease cancer progression, migration, and tumor size in several malignancies [41]. With this background, we used KPT-9274, a novel dual inhibitor of PAK4 and NAMPT, to test its effects on NHL. There are several PAK4 inhibitors, including PF3758309, LCH 7749944, and Inka1. However, most of these agents target PAK4 kinase activity. On the other hand, KPT-9274 is unique in being an allosteric modulator, which reduces only the steady-state level of PAK4 protein in cells, independent of the kinase activity [42]. Additionally, KPT-9274 also inhibits NAMPT activity, subsequently decreasing the NAD/NADH ratio, which eventually prevents the cancer cells from meeting their metabolic needs. 

We observed a marked reduction in tumor size in the animal tumor models with minimal toxicity (negligible loss of body weight). Tumor analysis revealed a decrease in PAK4 expression, as well as its downstream effector β-catenin. The anti-apoptotic protein Bcl-2 also decreased significantly, while there was a slight increase in Caspase 9. In the systemic model, drug treatment eventually leads to an increase in survival and a dramatic decrease in tumor cell count in the blood smears of the treatment group. These results are similar to other studies where KPT-9274 was used to treat different cancer cell lines. For example, Rane et al. demonstrated that KPT-9274 mediated inhibition of cell proliferation in vitro and significantly reduced tumor volume in a xenograft model following oral administration without affecting the mice’s body weight [43]. A Phase 1 clinical trial (NCT02702492) evaluated the efficacy of KPT-9274 in patients with advanced solid malignancies or NHL. In agreement with our current findings, evaluable response and NAPRT1 dependent rescue with niacin were observed in patients on this trial [44]. This has allowed further room for dose escalation, which is currently ongoing. Furthermore, in a study evaluating niacin co-administration with NAMPT inhibitors, significant loss of in vivo efficacy has been reported in NAPRT1-deficient cell culture- and patient-derived xenograft models [35]. In our study of MCL tumor xenograft models we have observed that KPT-9274 treatment caused a significant reduction in tumor volumes and a co-dosing of niacin with KPT-9274 did not result in loss of efficacy. These results are encouraging for the bedside, where supplementing niacin may alleviate the adverse effects without compromising the efficacy of the treatment.

## 5. Conclusions

In summary, we have demonstrated potent anti-tumor activity of a PAK4-NAMPT dual inhibitor, KPT-9274, in preclinical models of NHL. Our findings have established PAK4 and NAMPT as novel therapeutic targets in DLBCL, FL, and MCL. Further clinical investigations are warranted to establish its translational relevance for NHL patients. 

## Figures and Tables

**Table 1:** Sequences of primers used.

Primers	Sequences	
*PAK4*	Forward	GTGCAAGAGAGCTGAGGGAG
	Reverse	ATGCTGGTGGGACAGAAGTG
*Bcl-2*	Forward	TGAACTGGGGGAGGATTGTG
	Reverse	CGTACAGTTCCACAAAGGCA
*β-Catenin*	Forward	CGCCATTTTAAGCCTCTCGG
	Reverse	CTCCTCAGACCTTCCTCCGT
*Caspase 9*	Forward	TGTTCAGGCCCCATATGATCG
	Reverse	CAACTTTGCTGCTTGCCTGT
*β-actin*	Forward	GCACAGAGCCTCGCCTT
	Reverse	TCATCATCCATGGTGAGCTG
*18S*	Forward	GCAATTATTCCCCATGAACG
	Reverse	GGCCTCACTAAACCATCCAA

## Data Availability

The data presented in this study are available on request from the corresponding author.

## Supplementary Materials

The following are available online at https://www.mdpi.com/article/10.3390/cancers14010160/s1, Figure S1: PAK4 immunohistochemistry on NHL tissue sections, Figure S2: Effect of KPT-9274 on normal cells, Figure S3: KPT-9274 induces cell cycle arrest in WSU-FSCCL cells, Figure S4: Full Western Blot Figures, Table S1: Pathology report for tissue sections.
