# Supplementary material for: PAK4 and NAMPT as Novel Therapeutic Targets in Diffuse Large B-Cell Lymphoma, Follicular Lymphoma, and Mantle Cell Lymphoma
Source: Cancers (Basel). 2021 Dec 29;14(1):160. doi: 10.3390/cancers14010160 (PMC8750170; doi:10.3390/cancers14010160)
Supplement: Supplementary file 1 [file cancers-14-00160-s001.zip › cancers-1337506-supplementary.pdf]

Supplementary Files

PAK4 and NAMPT as Novel Therapeutic Targets in Diffuse Large B-Cell Lymphoma, Follicular Lymphoma, and Mantle Cell Lymphoma

Husain Yar Khan, Md. Hafiz Uddin, Suresh Kumar Balasubramanian, Noor Sulaiman, Marium Iqbal, Mahmoud Chaker, Amro Aboukameel, Yiwei Li, William Senapedis, Erkan Baloglu Ramzi M. Mohammad, Jeffrey Zonder and Asfar S. Azmi

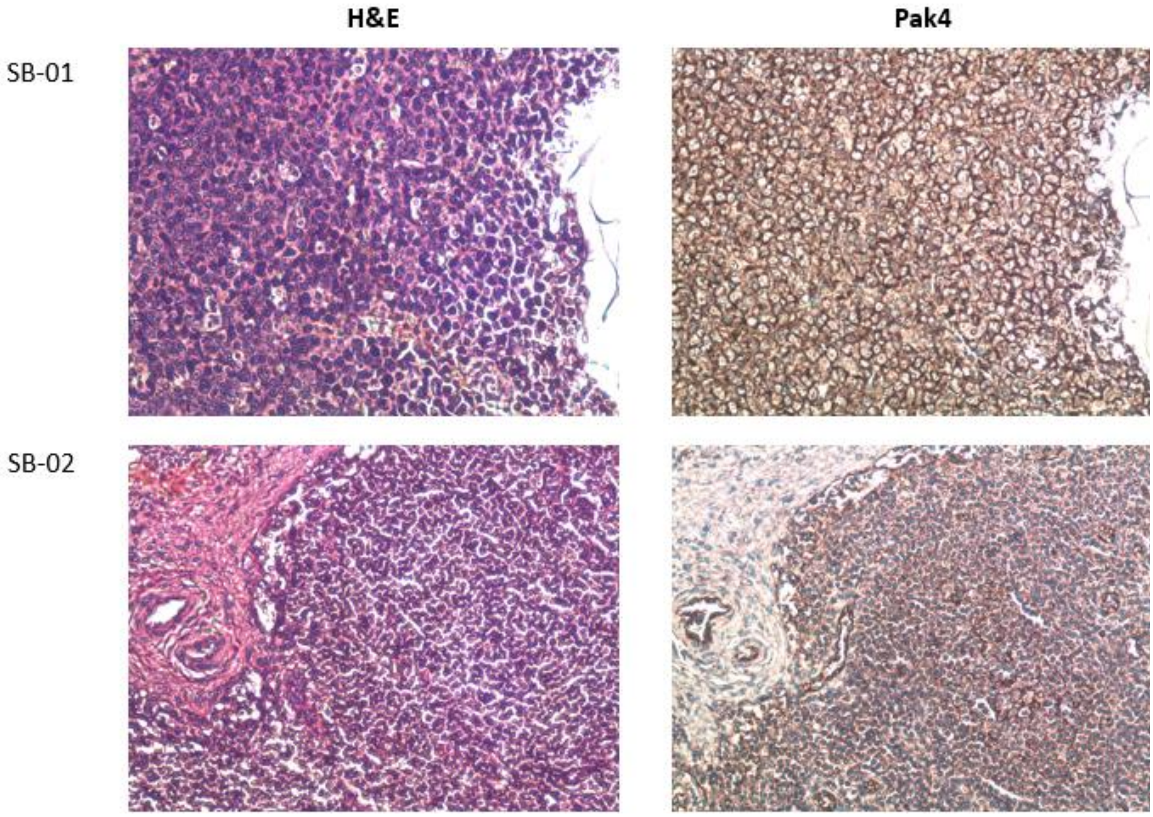

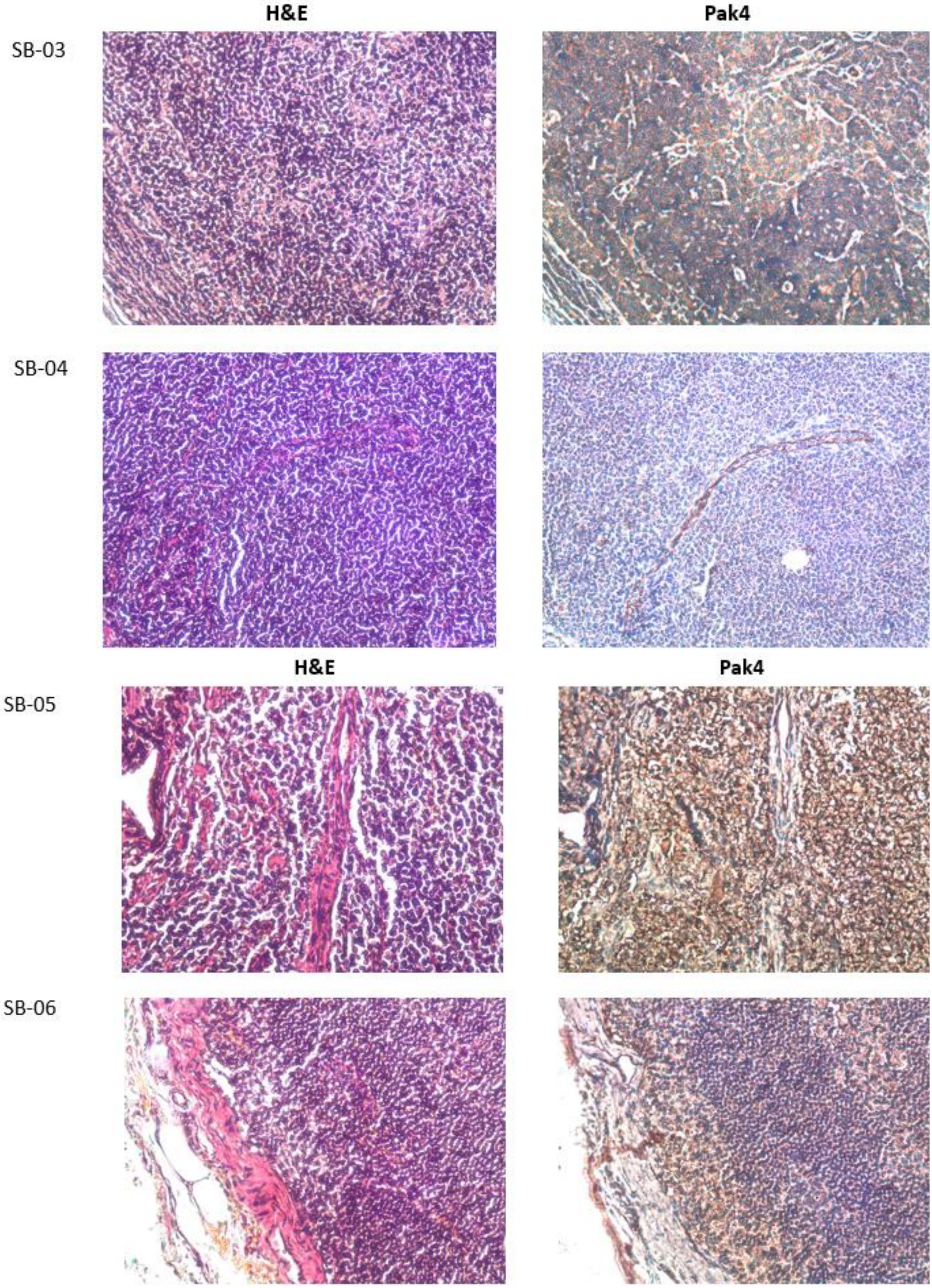
H&E
Pak4
SB-03
SB-04
H&E
Pak4
SB-05
SB-06

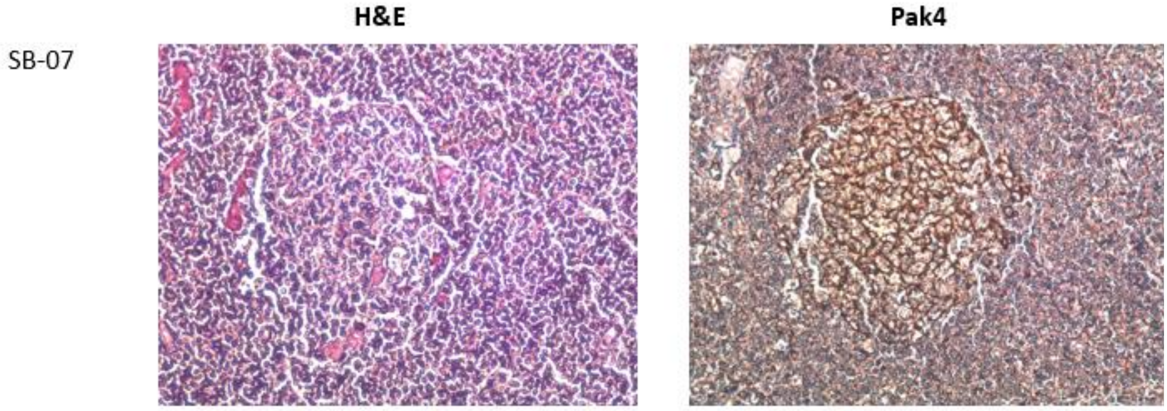

Figure S1. PAK4 immunohistochemistry on NHL tissue sections. IHC was performed on seven NHL specimen (SB-01–SB-07) that were obtained from the biorepository of Karmanos Cancer Institute. Briefly, the paraffin embedded tissues sections were stained with anti-PAK4 antibody obtained from Origene (Rockville, MD, USA) at a dilution of 1:200. The images were captured at 200× magnification using a light microscope and staining scores were estimated using the method described in section 2.2.

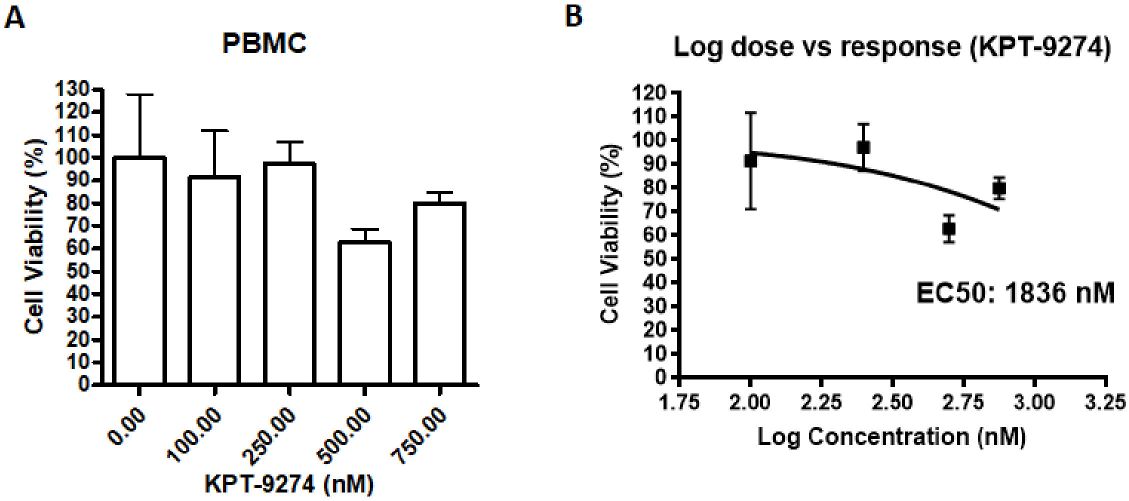

Figure S2. Effect of KPT-9274 on normal cells. 2 × 105 peripheral blood mononuclear cells (PBMC) were seeded in duplicates in 24 well plates and exposed to different concentrations of KPT-9274 for 72 h. At the end of the treatment period, cell viability was determined by Trypan Blue dye exclusion method and four values per treatment were plotted (**A**). A log dose vs response curve was generated using GraphPad Prism to obtain the EC50 (**B**).

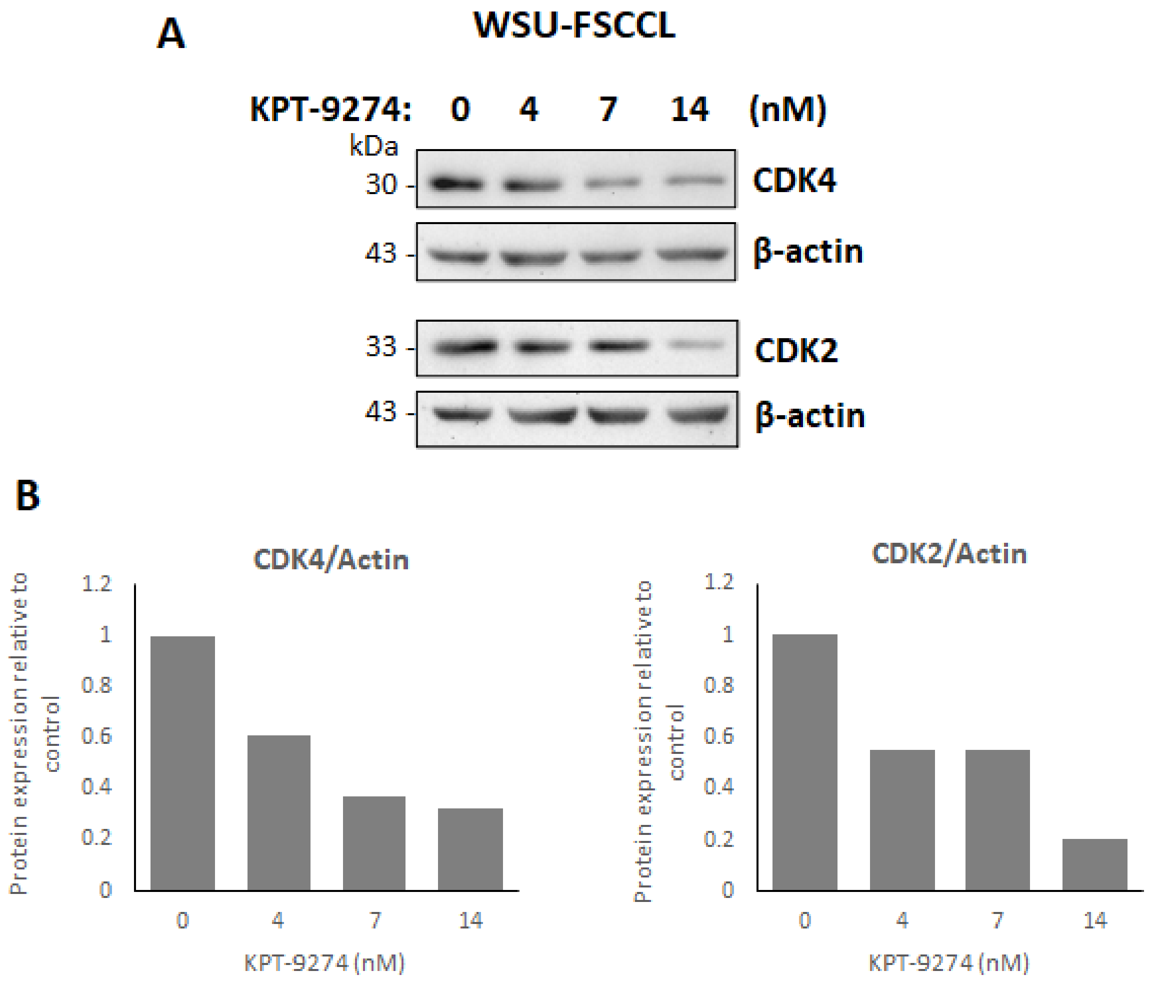

Figure S3. KPT-9274 induces cell cycle arrest in WSU-FSCCL cells. WSU-FSCCL cells were treated with indicated concentrations of KPT-9274 for 72 hrs. At the end of the treatment, cells were centrifuged, and protein was isolated using standard procedures. Equal amounts of proteins were resolved on 10% polyacrylamide gels, followed by Western Blotting. (**A**) The blots were probed for cell cycle proteins. β-actin was used as a loading control. (**B**) Change in protein expression was quantified by measuring relative band intensities using NIH ImageJ 1.5Oi software.

Figure 2C

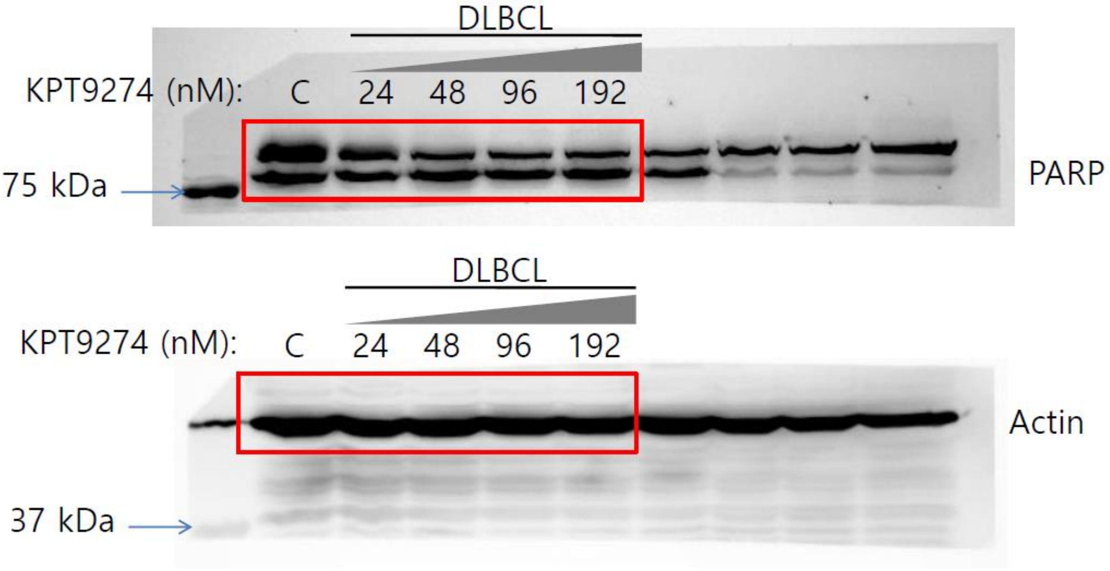

Figure 2C

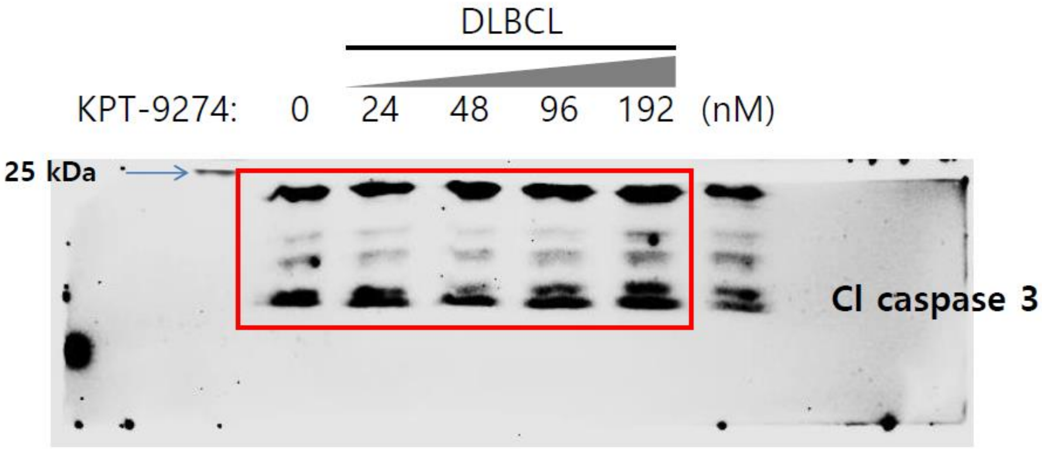

Figure 2C

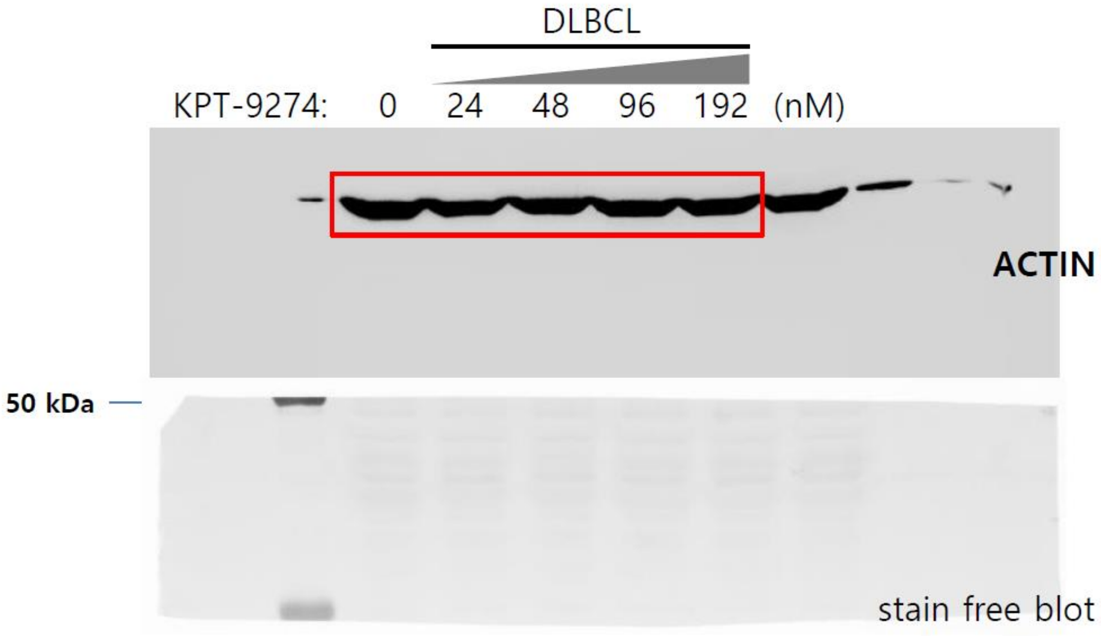

Figure 2C

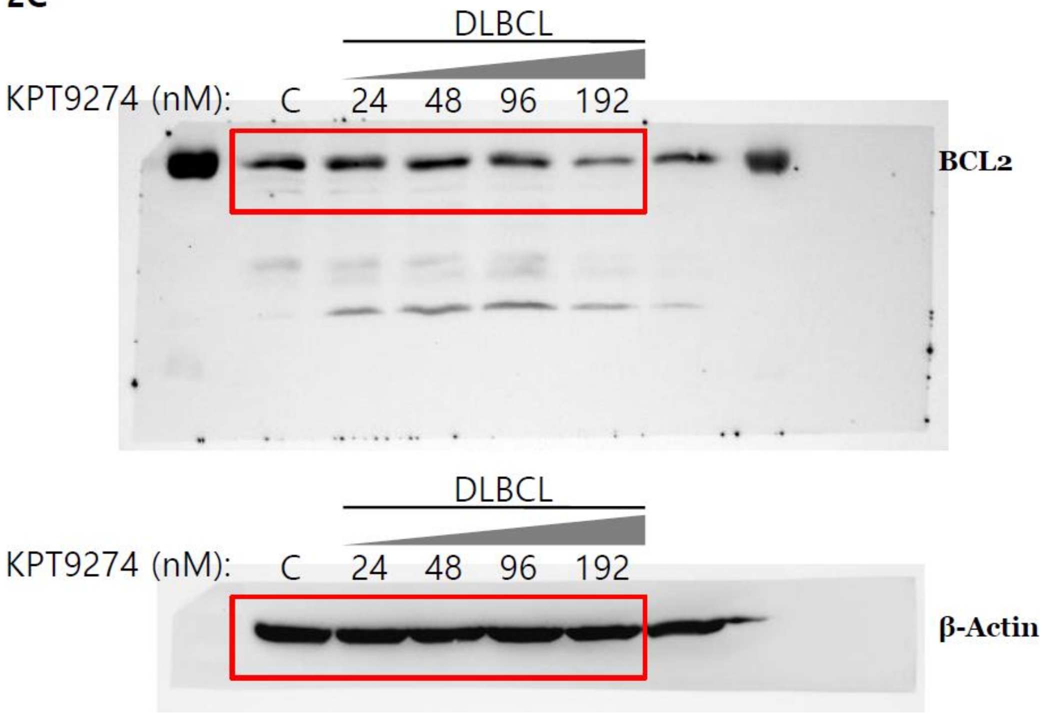

Figure 2C

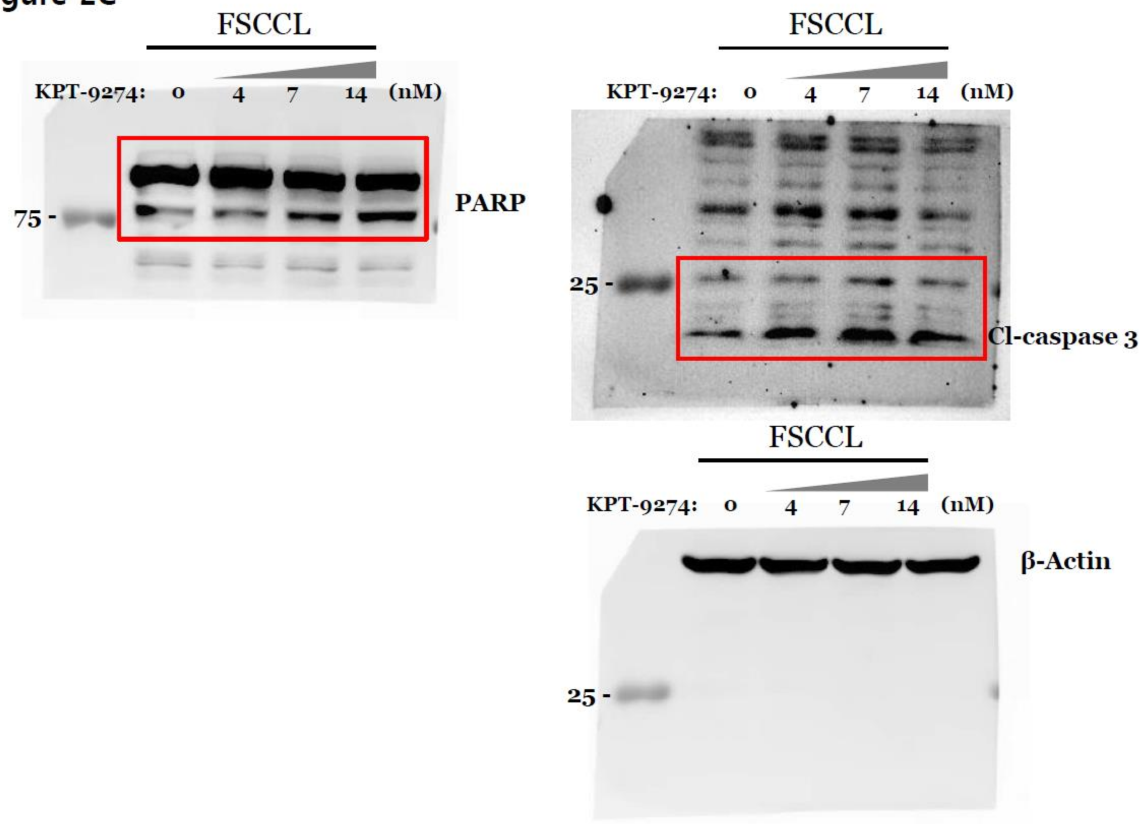

Figure 2C

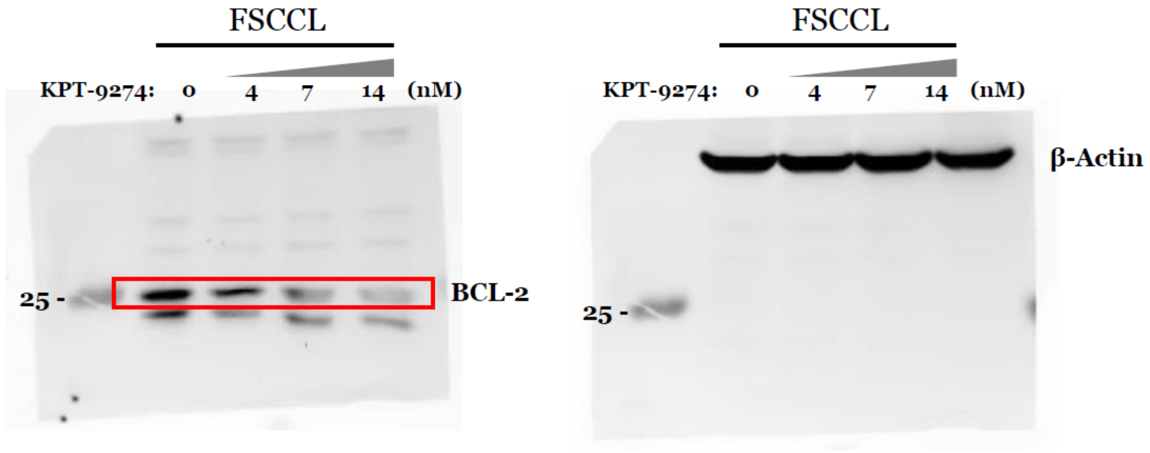

Figure 5D

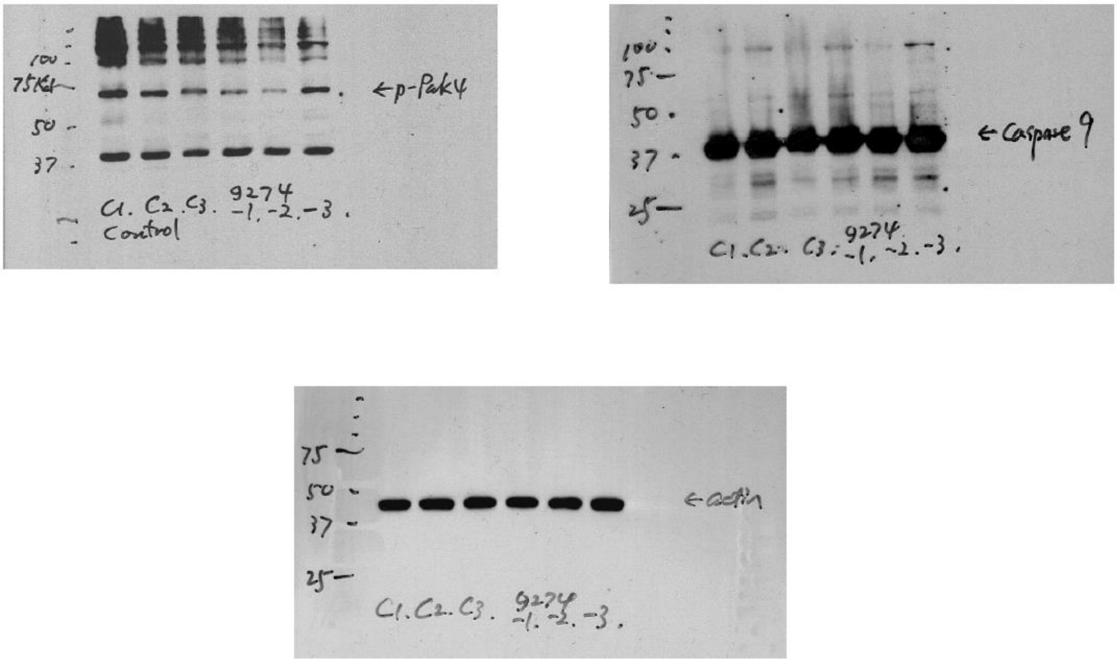

Figure S2A

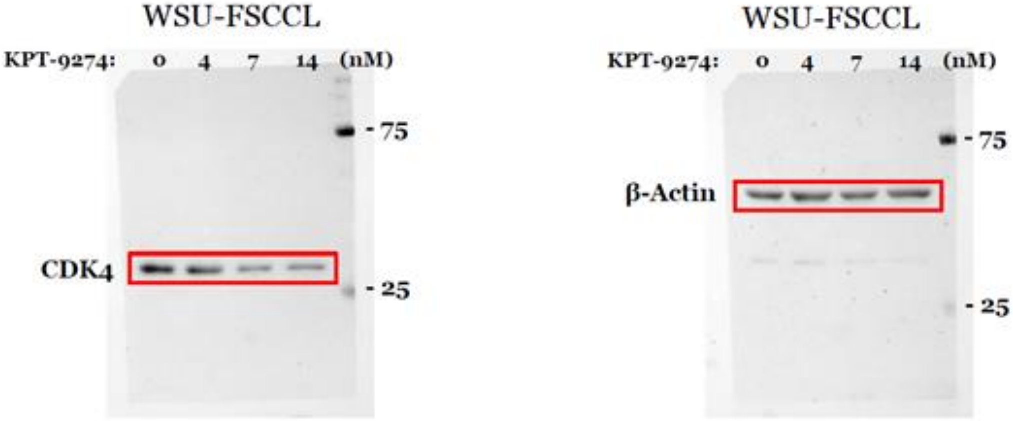

Figure S2A

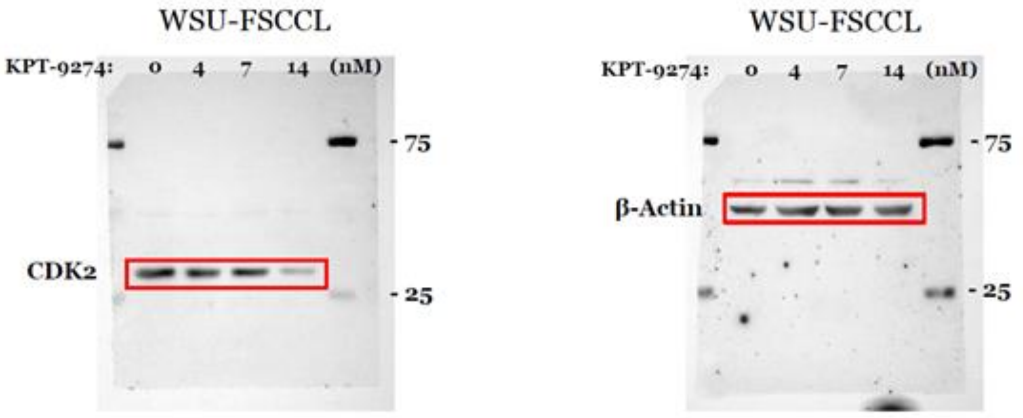

Figure S4. Full Western Blot Figures.

Table S1. Pathology report for tissue sections:

Specimen	Original Pathology	Staining Score	+ (%)	++ (%)	+++ (%)
SB-01	DLBCL (GC subtype)	130	30	50	
SB-02	DLBCL (ABC subtype) (Ki67 90%)	122	60	28	2
SB-03	Low grade follicular center NHL (Ki67 30%)	11	5	3	
SB-04	Low grade NHL (Ki67 30%)	6	6		
SB-05	DLBCL (ABC subtype) (Ki67 90%)	135	45	30	10
SB-06	Small lymphocytic lymphoma	28	8	10	
SB-07	Mantle cell lymphoma (Ki67 80%)	175	30	35	25
